# Retraction: Elevation of USP4 antagonizes oxygen glucose deprivation/reoxygenation-evoked microglia activation and neuroinflammation-mediated neurotoxicity *via* the TRAF6-NF-κB signaling

**DOI:** 10.1039/d1ra90021h

**Published:** 2021-01-21

**Authors:** Laura Fisher

**Affiliations:** Royal Society of Chemistry Thomas Graham House, Science Park, Milton Road Cambridge CB4 0WF UK advances-rsc@rsc.org

## Abstract

Retraction of ‘Elevation of USP4 antagonizes oxygen glucose deprivation/reoxygenation-evoked microglia activation and neuroinflammation-mediated neurotoxicity *via* the TRAF6-NF-κB signaling’ by Zhaoxia Wang, *RSC Adv.*, 2019, **9**, 23916–23924, DOI: 10.1039/C9RA03614H.

The Royal Society of Chemistry hereby wholly retracts this *RSC Advances* article due to concerns with the reliability of the data. The images in the article, and the raw data provided by the authors, were screened by an image integrity expert.

The expert was able to confirm that the raw data provided for the western blots in Fig. 1D, Fig. 2B, Fig. 2E, Fig. 5A and Fig. 5D showed evidence of manipulation, most likely in order to hide additional blots that do not appear in the published figures. Therefore, the raw data provided by the authors cannot be used to validate the published data.

In addition, there is a repeating section of cells duplicated within the microglia panel in Fig. 1A, which implies that the image has been manipulated.

Given the significance of the concerns about the validity of both the data in the article and the raw data provided by the authors, the findings presented in this paper are not reliable.

The authors have been informed but have not responded to any correspondence regarding the retraction.

Signed: Laura Fisher, Executive Editor, *RSC Advances*

Date: 7^th^ January 2021

## Supplementary Material

